# The emerging coloprotective effect of sildenafil against ulcerative colitis in rats via exerting counterbalance between NF-κB signaling and Nrf-2/HO-1 pathway

**DOI:** 10.1007/s10787-022-01016-9

**Published:** 2022-07-14

**Authors:** Ghada S. El-Tanbouly, Rehab S. Abdelrahman

**Affiliations:** 1 Department of Pharmacology, Faculty of Pharmacy, Delta University for Science and Technology, Gamasa, 11152 Egypt; 2 Department of Pharmacology and Toxicology, Faculty of Pharmacy, Mansoura University, Mansoura, 35516 Egypt; 3 Department of Pharmacology and Toxicology, College of Pharmacy, Taibah University, Al-Madina Al-Munawwarah, Medina, 30001 Saudi Arabia

**Keywords:** Sildenafil, Acetic acid, Ulcerative colitis, NF-κB p65, Nrf-2, HO-1

## Abstract

The current work explored the influences of time dependent Sildenafil (SILD) administration, and the possible outcomes from its concomitant administration with dexamethasone against acetic acid-induced ulcerative colitis in rats. Rats were assigned into six random groups: diseased group (AA), injected once with 2 ml acetic acid (3%) intrarectally, 2 days before sacrification. SILD + AA, received sildenafil (25 mg/kg, orally) for 6 days starting 3 days pre-injection of AA; SILD-t + AA, received sildenafil (25 mg/kg, orally), starting at time of AA injection and continued for 3 days; DEXA + AA, received dexamethasone (2 mg/kg, i.p.) for 3 days, starting at time of AA injection; SILD-t + DEXA + AA, received sildenafil (25 mg/kg, orally) and dexamethasone (2 mg/kg, i.p.), as mentioned. Sildenafil markedly ameliorated disease activity index (DAI), ulcer scores, colon length shortening and colonic histopathological changes. Mechanistically, Sildenafil markedly attenuated immunoexpression of NF-κB p65/ TNF-α and COX-2, diminished oxidative stress (↓ MDA/NO levels and ↑ GSH level and SOD activity), increased levels of Nrf-2/HO-1, compared to untreated group. Taken together, Sildenafil treatment suppressed acetic acid-induced ulcerative colitis, probably via inhibiting NF-κB/TNF-α signaling dependent of Nrf-2/HO-1 pathway, reducing oxidative stress and attenuating inflammation. Surprisingly, effects of sildenafil were unpromoted in a time dependant manner. Short term treatment with sildenafil was sufficient to exert its coloprotective effect, while longer term pretreatment was only superior among other treatments in the macroscopical changes. Moreover, concurrent administration of sildenafil and dexamethasone had the preference in boosting the antioxidant defense and anti-inflammatory mechanisms, visualized by histopathological/immunohistochemical changes.

**Electronic supplementary material:**

The online version of this article (10.1007/s10787-022-01016-9) contains supplementary material, which is available to authorized users.

## Introduction

Ulcerative colitis (UC) is a recurrent, chronic inflammatory disease of the colon of unknown etiopathogenesis at present (Brown and Mayer 2007). There is convincing evidence that excessive inflammation and oxidative stress are extremely involved in its pathogenesis (Jena et al. 2012). Acetic acid-induced UC animal model, is optimum as it resembles UC in humans in pathology, clinical course, increased release of inflammatory mediators, localized involvement and damage to the inflamed mucosa (Randhawa, Singh et al. 2014).

Nuclear erythroid-related factor 2 (Nrf-2) is a key antioxidant-mediated transcription factor that regulates downstream cytoprotective pathways, through interconnection with antioxidant response elements (ARE), which are localized upstream enhancer area of detoxification enzyme genes (Venugopal and Jaiswal 1998). Heme oxygenase-1 (HO-1) is one of the antioxidant enzymes, elevated following oxidative stress. HO-1 initiates the oxidative degradation of heme to carbon monoxide and bilirubin, both are potent antioxidants (Clark, Foresti et al. 2000). The activity of the transcription factor Nrf-2 is suppressed by Kelch-like ECH-associated protein-1 (KEAP-1), which physically segregates Nrf-2 in the cytosol, while electrophilic and oxidative stress destabilized this connection. Nrf-2 is then transfered to the nucleus, where it triggers downstream cytoprotective genes like HO-1 to be transcribed. Therefore, drugs that target Nrf-2 may be able to alleviate UC.

Nuclear erythroid-related factor 2 (Nrf-2) is greatly implicated in the transcription of genes responsible for management of cellular anti-inflammatory and antioxidant machinery (Ma 2013). Increased oxidative stress in absence of Nrf-2 induces NF-κB-over mediated cytokine expression, since NF-κB is more readily generated in an oxidative media (Ganesh Yerra, Negi et al. 2013). Nuclear factor kappa B (NF-κB) is a heterodimer protein complex constitute diverse subunits including P65 (Lingappan 2018). NF-κB signaling, and its downstream production of various proinflammatory cytokines, cyclooxygenase-2 (COX-2) enzyme, nitrosative and oxidative stress, perform a vital part in the pathogenesis of UC. These factors interact together, leading to exaggerated inflammatory reactions, thus contributed to colonic injury in UC (Jobin and Sartor 2000; Surh et al. 2001).

Overproduction of COX-2 due to disturbances in arachidonic acid metabolic pathway is well related to colitis development. COX-2 triggers overproduction of prostaglandin E2 (PGE-2) and thromboxane B2 (TXB2), which are two of the vital inflammatory mediators in colitis-associated colonic cell damage, inflammation, and edema, in addition to TXB2-induced vasoconstriction, platelet aggregation, and thrombosis (Dong et al. 2003) Previous studies confirmed their role in UC pathogenesis in animal models of colitis (Auwerda et al. 2001, Medina 2001, Appleyard et al. 2002).

Sildenafil (SILD) is a potent selective inhibitor of PDE5 and is widely being used for the treatment of erectile dysfunction in men (Yaseen, Darwich et al. 2012). More recently it has been experimented for treating other diseases. SILD is known to significantly decrease oxidative stress and inflammation in several models (Ali et al. 2011, El-Mahdy et al. 2016, LUNA 2016, Mohey et al. 2016, Sikandaner et al. 2017), thus yields a remarkable anti-inflammatory and antioxidant properties. It was thought of interest to examine the power of SILD to mitigate the effects of acetic acid-induced UC. In this research, we postulated that SILD could suppress UC in rats via Nrf-2/HO-1 pathway. Therefore, the current study was designed to compare three different regimens of SILD with or without dexamethasone to detect the most effective regimen.

## Materials and methods

### Drugs and chemicals

Sildenafil (Sild) was purchased from (Pfizer, Egypt) and was dissolved in water. Acetic acid was purchased from (EL-Goumhouria Co. for trading medicines, Egypt) and Dexamethasone was purchased from (AMRIYA pharmaceuticals, Egypt). These drugs were dissolved in normal saline. Other chemicals were of high analytical grade.

### Animals

Adult male SD rats weighting 250 ± 20 g were maintained in a room at standard conditions and were provided food and water ad libitum.

### Experimental design

**Fig. 1:**
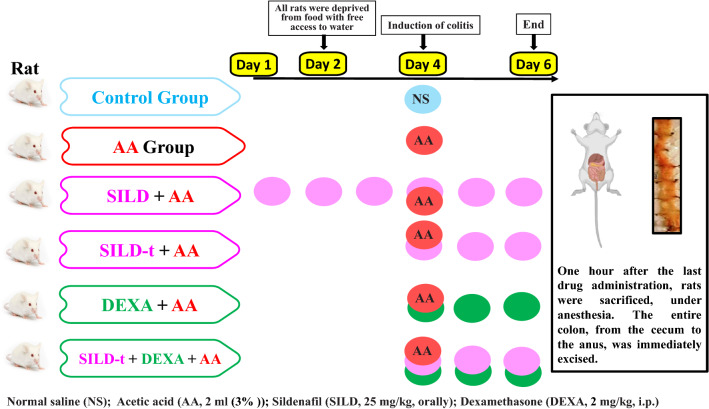
Schematic presentation of the experimental design

The experimental design is shown in Fig. 1. Rats were assigned randomly into 6 groups (*n* = 9): Control, received 2 ml normal saline intrarectally at the induction day; diseased group (AA), injected once with 2 ml acetic acid (3%) intrarectally, 2 days before sacrification to induce colitis; SILD + AA, received sildenafil (25 mg/kg, orally) for 6 days starting 3 days pre-injection of AA; SILD-t + AA, received sildenafil (25 mg/kg, orally), starting at the time of AA injection and continued for 3 days; DEXA + AA, received dexamethasone (2 mg/kg, i.p.) for 3 days, starting at the time of AA injection; SILD-t + DEXA + AA, received sildenafil (25 mg/kg, orally) and dexamethasone (2 mg/kg, i.p.), for 3 days, starting at the time of AA injection. This dose of 25 mg/kg of SILD showed beneficial effect in several studies (Lee et al. 2010; Jasińska-Stroschein et al. 2013, Huang, Liu et al. 2022), in addition to optimal coloprotective effect according to our preliminary experiment (where two doses of sildenafil 25 and 30 mg/kg were used, supplementary file). All rats were deprived from food with free access to water 48 h before induction of colitis.

At the time of the experiment, anesthesia was achieved by thiopental (40 mg/kg, i.p.). Colitis was induced according to a published protocol (Matuszyk et al. 2016) by slow rectal injection of a 2 ml diluted solution of acetic acid (3%) using a flexible plastic rubber catheter with an outside diameter of 8 mm inserted rectally 8 cm into the colon via the anus. The animals were maintained in the supine trendelenburg position for 30 s to prevent early leakage of the intracolonic instillation.

One hour after the last drug administration, rats were sacrificed, under anesthesia. The entire colon, from the cecum to the anus, was immediately excised and the colon length was measured and observed as an indirect marker of inflammation, then washed, opened longitudinally and digital pictures were taken.

Finally, colon was cut into two parts, one part was homogenized in phosphate buffer solution (PBS), then homogenates were centrifuged at 4000 × g at 4 °C for 15 min. The supernatant was kept in − 80 °C until needed for subsequent analyses. The other part of colon was fixed in 10% buffered formalin for histopathological and immunohistochemical analyses.

### Assessment of disease activity index (DAI)

Animals were kept in cages and were observed for body weight change, stool consistency and bleeding for 2 days post induction of colitis, to score the disease activity index (DAI) using a protocol previously described by (Cooper et al. 1993) with little modification, as shown in Table 1.

**Table 1:** Scoring of disease activity index (DAI)

Weight loss	Stool consistency	Bleeding
0: < 1%	0: normal	0: normal
1: 1–5%	1: normal	1: occult blood ( +)
2: 5–10%	2: loose stool	2: occult blood (+ +)
3: 10–15%	3: loose stool	3: occult blood (+ + +)
4: > 15%	4: diarrhea	4: gross bleeding

The scores from these three parameters were summed to calculate the DAI, ranging from 0 (healthy) to 12 (maximal severity of colitis)

### Assessment of macroscopic ulcer score

Colons were examined visually, to score the ulceration. The damage was assessed by the colon mucosal damage index (CMDI) scored on a scale of 0–10 according to a reported scoring system (Bell et al. 1995).

The following scores were applied: Grade 0: normal appearance; Grade 1: focal hyperemia, no ulcers; Grade 2: ulcer with no significant inflammation (hyperemia and bowel wall thickening); Grade 3: ulcer with inflammation at one site; Grade 4: two or more sites of ulceration and inflammation; Grade 5: major sites of damage extending > 1 cm along the length of the colon; and Grade 6–10:, if major sites of damage extending > 2 cm along the colon, the score was increased by 1 for each additional cm of involvement.

### Estimation of antioxidant status and nitrosative stress

Levels of malondialdehyde (MDA), reduced glutathione (GSH) and superoxide dismutase (SOD) activity were assessed spectrophotometrically according to methods described by Ohkawa et al. (1979), Ellman (1959) and Marklund and Marklund (1974) respectively.

### Evaluation of the antioxidant-related markers (Nrf-2, HO-1) by ELISA assay

Levels of the oxidant-related marker: iNOS and levels of the antioxidant-related markers: nuclear erythroid-related factor 2 (Nrf-2) and heme oxygenase-1(HO-1) were evaluated in colon homogenate by ELISA assay using commercial kits obtained from (Bioassay Technology Laboratory, Shanghai Crystal Day Biotech Co., Ltd., Shanghai, China), according to manufacturer’s instructions.

Briefly, a specific biotinylated antibody reagent was added to the plates, which were filled with the homogenized tissue and incubated at 37^∘^C in CO_2_ for 2 h. After washing with phosphate-buffered saline (PBS), streptavidin horseradish peroxidase (HRP) solution was added, and the plates were incubated for 30 min at room temperature. Then, using a microplate reader, absorbance was measured at 450 nm.

### Immunohistochemical examination

Antibodies were used to evaluate colon expression of tumor necrosis factor alpha (TNF-α) (ABclonal Technology, MA, USA, catalog number: A0277), nuclear factor kappa beta p65 (NF-κB p65) (Bioss antibodies, MA, USA, catalog number: bs-20159R), and COX-2 (ABclonal Technology, MA, USA, catalog number: A1253), using Avidin–Biotin Complex (ABC), immunohistochemical staining protocol, according to the manufacturer's instructions.

Sections (4 μm) of colons were incubated for 20 min in 0.3% H2O2 to block the endogenous peroxidase activity. The section slides were incubated in a 100 °C water bath for 10 min in 0.01 M PBS buffer solution, then blocked with 2% BSA for 30 min. Then, the sections were incubated with the anti-rat antibodies overnight at 4 °C. The slides were washed and then incubated with HRP secondary antibody at 37 °C for 1 h. The sections were subsequently treated with DAB working solution for 4 min and finally counterstained with Mayer’s hematoxylin. Tissue sections were observed, photographed with a microscope (400 × magnification) and were semi-quantified.

### Histopathological examination of colon tissues

The colons were dissected and fixed with 10% formaldehyde solution for 48 h. The tissues were embedded in paraffin wax and sliced (5 μm). The sections were stained with hematoxylin–eosin (H&E) and were evaluated blindly by the pathologist under a microscope (100 × magnification).

#### Statistical analysis

Results are presented as the mean ± standard error of the mean (SEM). Statistical evaluation was carried out by GraphPad Prism 5 (GraphPad Software, San Diego, CA, USA). One-way analysis of variance (ANOVA), followed by Tukey Kramer's post-hoc test was used to compare the measures between the experimental groups. Kruskal–Wallis test followed by Dunn's multiple comparison post-hoc test was used for analysis of histopathological and histochemical scores of rats. Significance level was set at *p* < 0.05.

## Results

### Effect of sildenafil on DAI, colon length, and macroscopic ulcer score

**Fig. 2:**
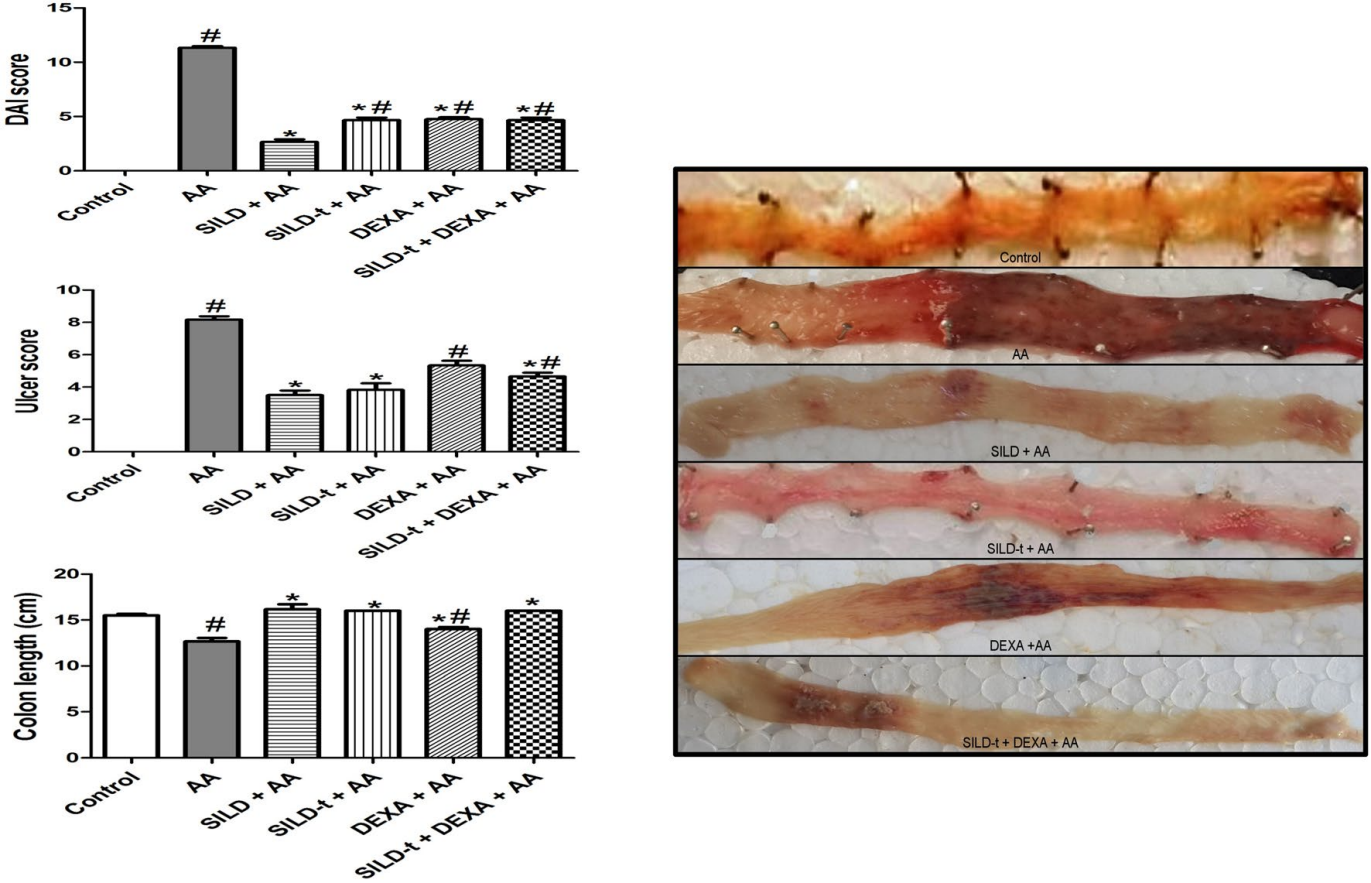
Effect of sildenafil on DAI, colon length, and macroscopic ulcer score. Data are expressed as mean ± SEM (*n* = 9) *AA* Acetic acid, *SILD* Sildenafil, *DEXA* Dexamethasone, *DAI* disease activity index. Statistically significant: # *p* < 0.05, compared to control group, *p < 0.05, compared to AA group, using one-way ANOVA followed by Tukey–Kramer multiple comparisons post-hoc test

Acetic acid treatment caused a significant increase in DAI and macroscopic ulcer score with a significant reduction in colon length compared to control group. DEXA + AA group significantly reduced intensity of DAI by 58% with a significant increase in colon length by 1.1-fold, and exerted insignificant decrease in ulcer score, compared to AA group. Meanwhile, other treated groups: SILD + AA, SILD-t + AA, and SILD-t + DEXA + AA showed a higher marked decrease in intensity of DAI by 76.4, 58.8, 58.8%, and ulcer score by 57.1, 53, 42.8%, respectively, with a more significant increase in colon length by 1.26-fold, compared to AA group (Fig. 2), indicating the highest preference for the longer term pretreatment with sildenafil over other treated groups; SILD > SILD-t > SILD-t + DEXA > DEXA, in order of improvement of all macroscopical features.

### Effect of sildenafil on antioxidant status and nitrosative stress

**Fig. 3:**
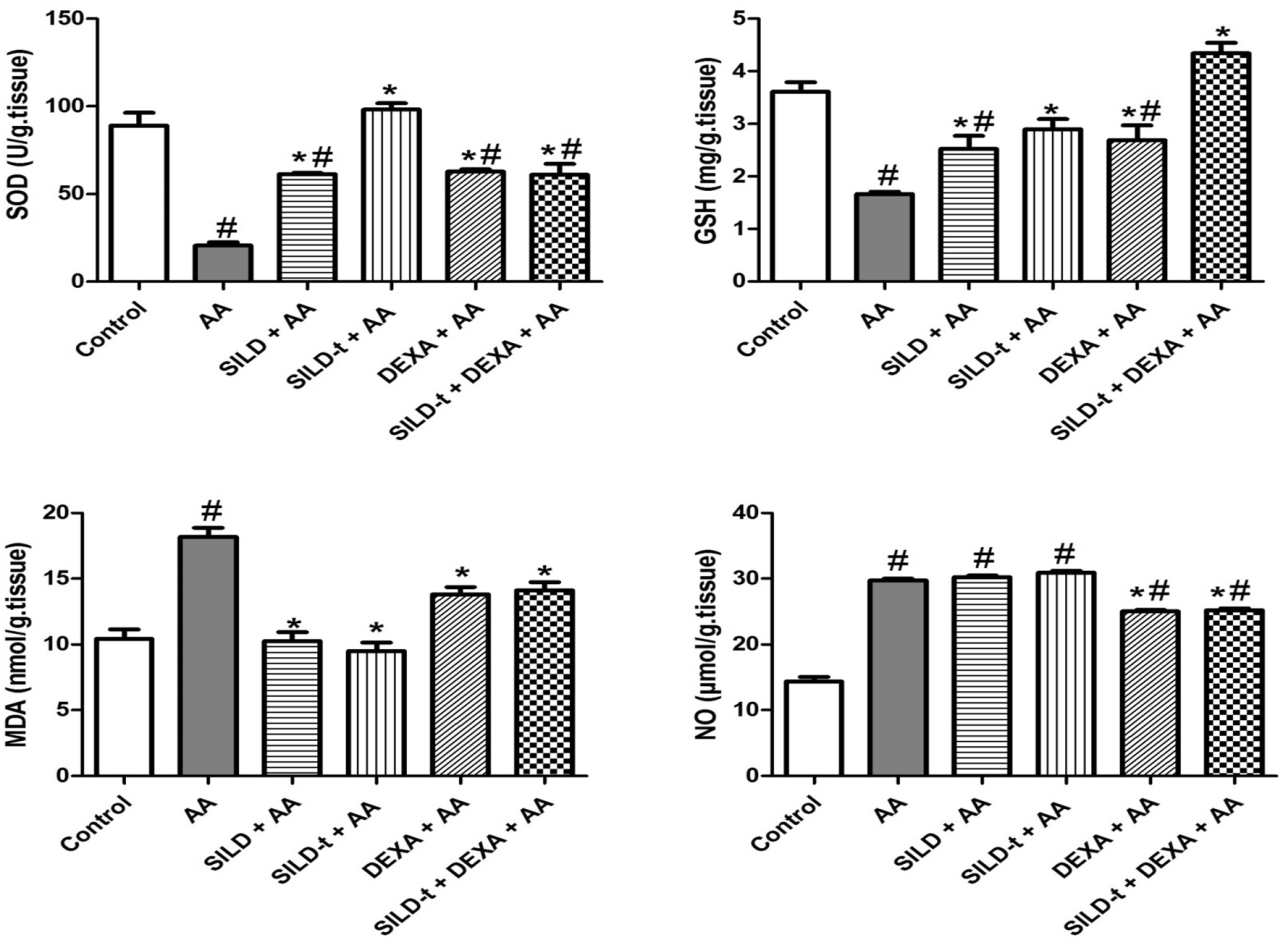
Effect of sildenafil on antioxidant status and nitrosative stress. Data are expressed as mean ± SEM (*n* = 9) *AA* Acetic acid, *SILD* Sildenafil, *DEXA* Dexamethasone, *MDA* malondialdehyde, *NO* nitric oxide, *GSH* reduced glutathione, *SOD* Superoxide dismutase. Statistically significant: #*p* < 0.05, compared to control group, *p < 0.05, compared to AA group, using one-way ANOVA followed by Tukey–Kramer multiple comparisons post-hoc test

To ascertain whether SILD can affect oxidative/nitrosative stress, we compared MDA, NO levels, SOD, and GSH activity within treated groups: SILD + AA, SILD-t + AA, DEXA + AA, and SILD-t + DEXA + AA (Fig. 3).

Acetic acid treatment caused a significant elevation in MDA and NO (by 1.7 and twofold, respectively), whereas SOD and GSH activity were significantly decreased (by 23% and 46%, respectively) when compared to control group.

Herein, uncombined dexamethasone treated group and longer term pretreatment with sildenafil produced the least significant effects among other treated groups. DEXA + AA group showed a significant increase in SOD activity and GSH level by 3 and 1.6 folds respectively, in addition to a significant decrease in MDA level by 24%. Meanwhile, it produced the most significant reduction in NO level by 15.7% compared to AA group, indicating that nitrosative stress can be highly enhanced with dexamethasone treatment.

SILD + AA group showed the least significant increase in SOD activity and GSH level by 2.9 and 1.5 folds respectively, however, its high significant reduction in MDA level by 43.6% among other groups indicated that lipid peroxidation can be highly enhanced in the pre-diseased state with this drug, whereas no change was recorded in NO level, compared to AA group.

Meanwhile, short term treatment with sildenafil alone or combined with dexamethasone produced the highest significant effects among other treated groups. SILD-t + AA and SILD-t + DEXA + AA groups showed a significant increase in SOD activity by 4.7 and 2.9 folds, respectively, in addition to a significant increase in GSH level by 1.7 and 2.6 folds, respectively. Moreover, they exerted a significant reduction in MDA level by 47.8, and 22%, respectively, in addition to a 15% significant decrease in NO level with the combined group, while no change was recorded in NO level in SILD-t + AA group, compared to AA group.

### Effect of sildenafil on expression of the antioxidant-related markers (NRF-2 and HO-1)

**Fig. 4:**
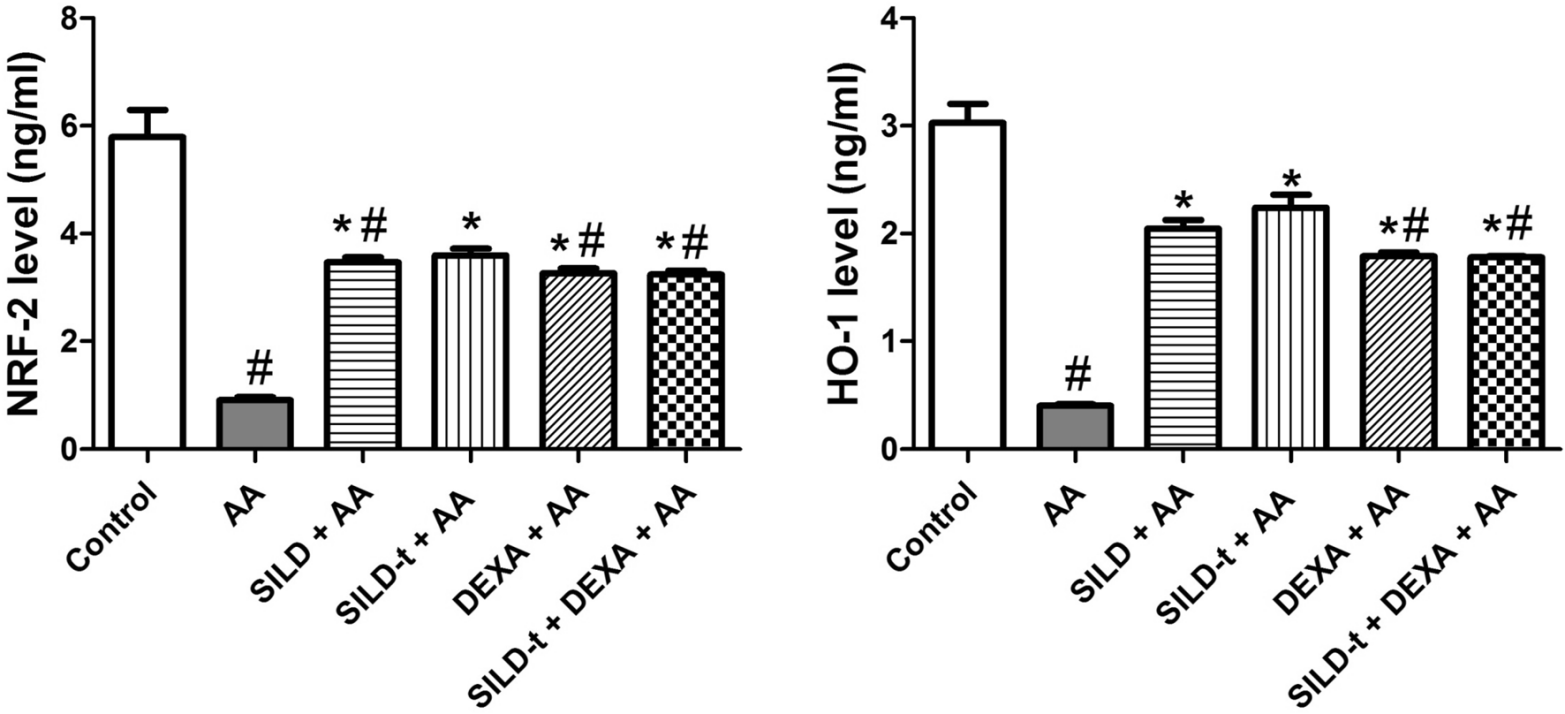
Effect of sildenafil on expression of the antioxidant-related markers (NRF-2 and HO-1). Data are expressed as mean ± SEM (*n* = 9) *AA* Acetic acid, *SILD* Sildenafil, *DEXA* Dexamethasone, *Nrf-2* Nuclear erythroid-related factor 2, *HO-1* Heme oxygenase-1. Statistically significant: #*p* < 0.05, compared to control group, *p < 0.05, compared to AA group, using one-way ANOVA followed by Tukey–Kramer multiple comparisons post-hoc test

In comparison to the normal control group, AA injection decreased NRF-2 and HO-1 levels by 15.7% and 13.3%, respectively) (Fig. 4). All treatment exerted a significant elevation in NRF-2 and HO-1 levels, compared to AA group, with the highest preference for time dependant sildenafil treatments, followed by single and combined DEXA treatments; SILD-t > SILD > DEXA and SILD-t + DEXA.

### Effect of sildenafil on immunohistochemical evaluation of the inflammatory mediators (TNF-α, NF-κB and COX-2)

**Fig. 5:**
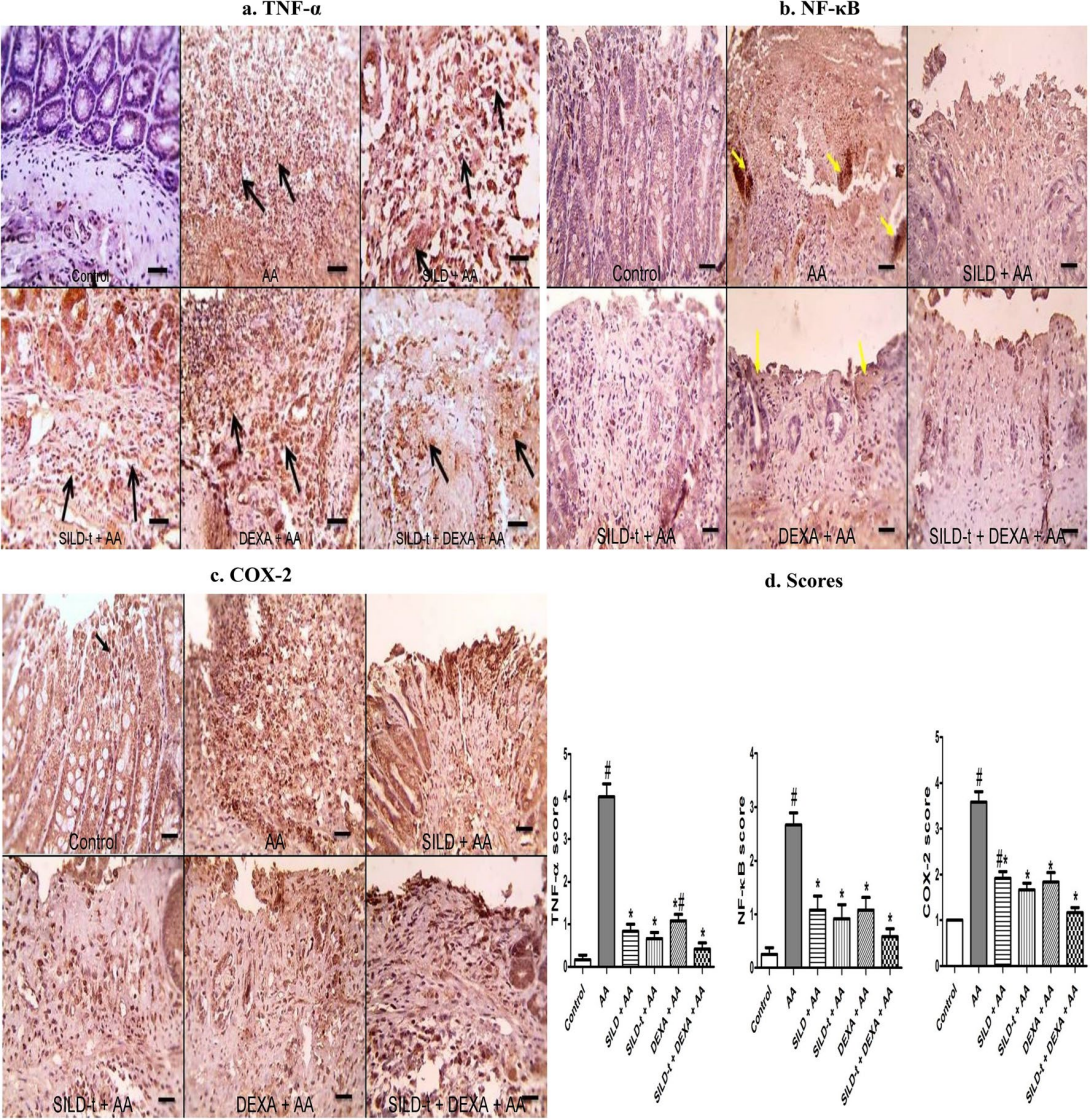
Effect of sildenafil on immunohistochemical evaluation of the inflammatory mediators (TNF-α, NF-κB and COX-2). **a **Tumor necrosis factor alpha (TNF-α), **b** Nuclear factor kappa B (NF-κB), **c** Cyclooxygenase-2 (COX-2) expression in colonic tissues assessed by immunohistochemistry (X 400, bar = 50 µm), **d** Bar graphs showing immunohistopathological score of TNF-α, NF-κB and COX-2 in colonic tissue of rats. Statistically significant: #*p* < 0.05, compared to control group, *p < 0.05, compared to AA group, using Kruskal–Wallis test followed by Dunn's multiple comparison test. *AA* Acetic acid, *SILD* Sildenafil, *DEXA* Dexamethasone

Microscopic picture of immunostained colonic sections against TNF-α showing weak expression in control group. Colonic TNF-α positive expression appear as brown cytoplasmic color in leukocytes (macrophages & neutrophils) infiltrating mucosa and submucosa and there was also a sparse distribution in epithelial cells and vascular endothelial cells. The numbers of positively stained cells were predominantly located within the mucosa and submucosa and were significantly decreased in all treated group. The positive reaction appears the lowest in SILD-t + DEXA + AA (Fig. 5a).

Microscopic pictures of immunostained colonic sections against NF-κB showing normal expression in mucosa with negative expression in submucosa in control group. In contrast, intense positive staining appears in affected areas with necrosis & ulceration in mucosa (yellow arrows) and underlying submucosa (*) in AA group. Treated groups: SILD + AA, SILD-t + AA and DEXA + AA showed marked decrease of positive expression in mucosa (yellow arrows) with persistence of positive expression in submucosa (*). This decrease appeared the lowest in DEXA + AA group when compared to AA group. The treated group: SILD-t + DEXA + AA showed marked decrease of positive expression in mucosa (yellow arrows) and submucosa (*), when compared to AA group. IHC counterstained with Mayer's hematoxylin (Fig. 5b).

Microscopic pictures of immunostained colonic sections against COX-2 showing normal expression in mucosa (area in square) with negative expression in submucosa in control group. In contrast, intense positive staining appears in affected areas with necrosis & ulceration in mucosa (area in square) and underlying submucosa (*) in AA group. Treated groups showed lowered positive staining in affected mucosa (area in square) and underlying submucosa (*) when compared with AA group. The positive reaction appeared the lowest in SILD-t + DEXA + AA followed by SILD-t + AA followed by DEXA + AA followed by SILD + AA. IHC counterstained with Mayer's hematoxylin (Fig. 5c).

### Effect of sildenafil on histopathological examination of the colon

**Fig. 6:**
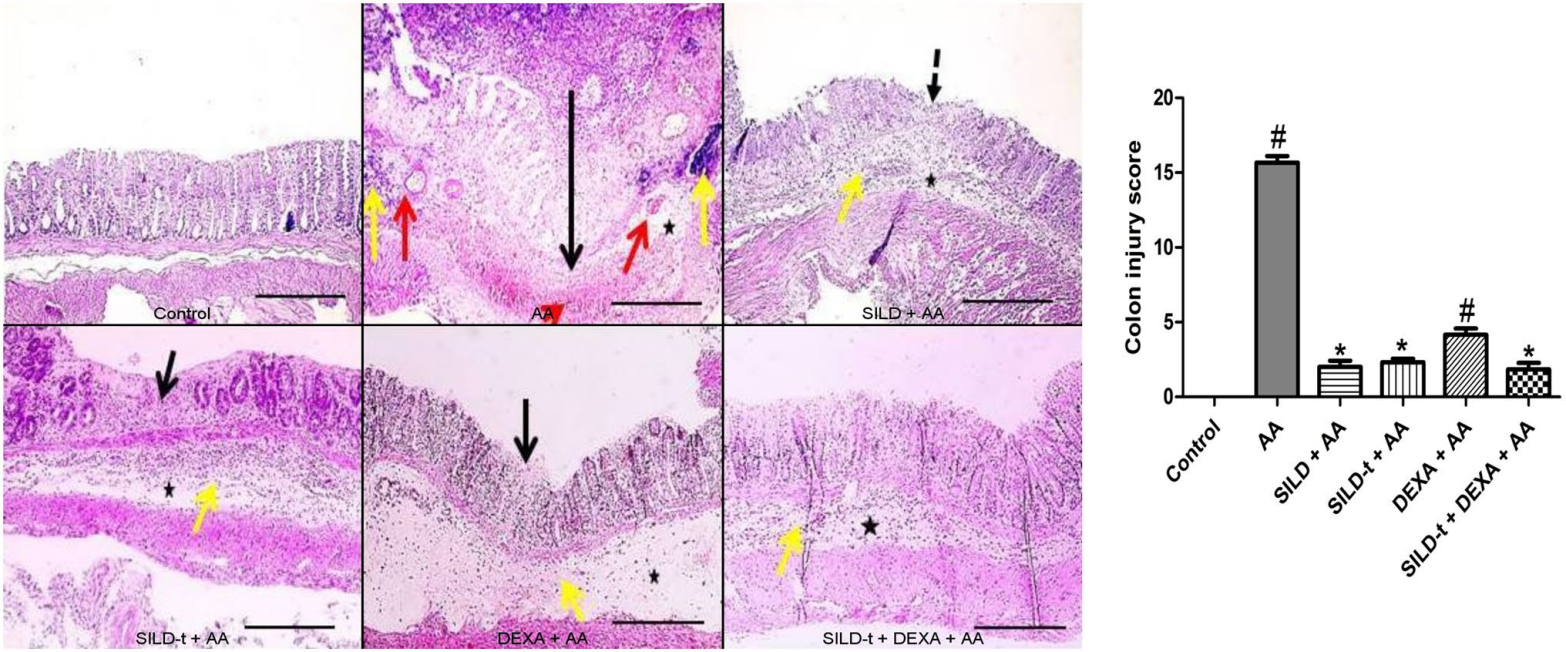
Effect of sildenafil on histopathological examination of the colon. Right panel**:** Representative photomicrographs of H&E-stained colonic sections (X 100, bar = 100 µm). Left panel: Bar graphs showing colon injury score. Statistically significant: #*p* < 0.05, compared to control group, *p < 0.05, compared to AA group, using Kruskal–Wallis test followed by Dunn's multiple comparison test. *AA* Acetic acid, *SILD* Sildenafil, *DEXA* Dexamethasone

Microscopic pictures of H&E-stained colonic sections showing normal layers: mucosa, submucosa and muscles in control group. Colonic sections from colitis group (AA) showed an extensive transmural necrosis reaching muscular layer, mucosal necrosis with ulceration accompanied with marked submucosal edema, congestion, hemorrhage and inflammation. Colonic sections from treated group (SILD + AA) showed mild focal mucosal erosion, markedly reduced submucosal edema, congestion and inflammation. Colonic sections from treated groups: (SILD-t + AA), followed by (DEXA + AA) showed small focal area of mucosal necrosis, submcosal edema and inflammation with few leukocytic cells infiltration. Colonic sections from treated group (SILD-t + DEXA + AA) showed markedly reduced submucosal edema and inflammation. Transmural necrosis (long black arrow), erosion/ulceration (dashed arrows), mucosal necrosis (short black arrow), submucosal edema (*), congestion (red arrow), hemorrhage (red arrowhead) and inflammation (yellow arrow) (Fig. 6).

## Discussion

**Fig. 7:**
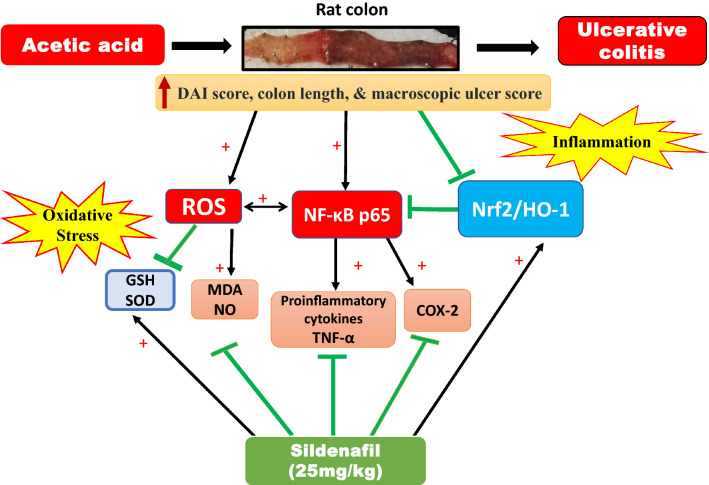
Graphical representation of the coloprotective mechanisms for Sildenafil against AA-induced UC in rats. ***AA*** Acetic acid, *SILD* Sildenafil, *DAI* disease activity index, *MDA* malondialdehyde, *NO* nitric oxide, *GSH* reduced glutathione, *SOD* Superoxide dismutase, *Nrf-2* Nuclear erythroid-related factor 2, *HO-1* Heme oxygenase-1, *TNF-α*: Tumor necrosis factor alpha, *NF-κB* Nuclear factor kappa B, *COX-2* Cyclooxygenase-2

AA-induced colitis is like human UC in cytokine profile and histopathological features and is characterized by neutrophil infiltration, that contributes substantially to the development of colon tissue damage via the formation of different reactive species (Elshazly, Elhassanny et al. 2020). Findings from this study show that the identified potent selective inhibitor of PDE5, sildenafil, attenuated AA-induced colitis in rat. SILD treatment produced the following beneficial effects: a) it improved macroscopic appearance of colon tissue and markedly reduced histopathological changes in colon tissue; b) it reduced colon oxidative stress (↓ MDA, NO levels and ↑ GSH level and SOD activity); c) it increased level of antioxidant-related markers Nrf-2 and HO-1; d) it decreased colon immunodepression of inflammatory markers TNF-α, NF-κB p65, and COX-2 (Fig. 7).

Nuclear erythroid-related factor 2 (Nrf-2) is a distinct antioxidant-mediated transcription factor that controls downstream cytoprotective targets, through encoded coordinated expression of antioxidant and cytoprotective genes, which is essential for body’s defense against inflammatory tissue injuries (He, Ru et al. 2020). Among which is HO-1, a significant cellular defense enzymatic antioxidant facing oxidative tissue damage that is considered as a protective mechanism through initiation of several antioxidants and bilirubin formation, which are capable of sequestering ROS (Bauer, Huse et al. 2008). Therefore, initiation of this enzyme could be a target to protect against inflammatory processes and oxidative tissue damage.

To pinpoint the exact mechanism of SLID. We investigated its role in modulating the Nrf-2/HO-1 pathway. The Nrf-2/ HO-1 pathway has been a recent attractive target in addressing AA-induced colitis (Khodir et al. 2019, Jeong, Kang et al. 2021). Indeed, in our study SILD treatment increased antioxidant-related markers Nrf-2 and HO-1, implying that it could be used to prevent oxidative damage and improve healing. Several studies reported antioxidant activities of SILD in experimental disease models through the Nrf-1/HO-1 cytoprotective pathway (Zahran et al. 2015, Behiry et al. 2018, Fang et al. 2020).

Inflammation is a major effector of AA-induced colitis. NF-κB is involved in the regulation of many genes involved in the inflammatory response (Liu et al. 2017). Increased NF-κB expression has been linked to increased cyclooxygenase-2 (COX-2) (Ahmed et al. 2014), TNF-α and IL-6 expression (Chamanara et al. 2019, Ansari et al. 2021), which are directly involved in colon tissue destruction. TNF-α may also increase the inflammatory cascade by activating NF-κB (Parameswaran and Patial 2010). Nrf-2 activators have been reported to be capable of preventing IK/IB phosphorylation and NF-κB p65 nuclear translocation, hence inactivating NF-κB signaling (Wardyn et al. 2015). Furthermore, it is thought that activating Nrf-2 antioxidant signaling inhibits inflammatory signaling pathways like NF-κB (Abdelrahman and Abdel-Rahman 2019). As a result, there is a possibility of crosstalk between the Nrf-2 and NF-κB pathways. In the current study AA-induced increase in inflammatory markers in line with previous studies (Elshazly et al. 2020, Ansari et al. 2021, Jeong et al. 2021, Salim et al. 2021), SILD reduced colon expression of TNF-α, NF-κB p65 and COX-2 consistent with previous studies (Yaseen et al. 2012, Zahran et al. 2015, Sikandaner et al. 2017, Fang et al. 2020).

Another point of view, AA-induced oxidative stress was indicated by significantly higher levels of NO and MDA, an index of lipid peroxidation, and significantly lower levels of the antioxidants GSH and SOD (Ansari et al. 2021, Salim et al. 2021, Zaghloul et al. 2022). SILD treatment has significantly reduced NO and MDA and elevated GSH and SOD levels and alleviated the oxidative stress status. SILD may potentially exert its action through potentiating the antioxidant Nrf-2 pathway. Moreover, SILD treatment reduced histopathological and score changes in colon tissue.

In addition, in this study, AA-induced UC which was manifested by a significant rise in DAI score, colon length, and macroscopic ulcer score that correlated with histopathological examination that revealed extensive transmural necrosis reaching muscular layer, mucosal necrosis with ulceration accompanied with marked submcosal edema, congestion, hemorrhage and inflammation. These results are in line with previous studies that confirmed the ability of AA to induce UC in rats closely comparable to the human UC ((Elshazly et al. 2020, Zaghloul et al. 2022). Interestingly, the administration of SILD significantly improved colon tissue architecture and reversed AA-induced UC.

Therefore, this work was the first to design a time dependent study on sildenafil, in addition to the concomitant sildenafil/dexamethasone effects. Three regimens: SILD/DEXA combination, short and longer term SILD treatments, were able to exert downregulation of inflammatory mediators, increased colon antioxidant defense machinery, with a preference for the combination group, while the longer term sildenafil pretreatment regimen was the least significant, except for macroscopical changes. In addition, the underlying coloprotective mechanisms of sildenafil were primarily explained in our study.

## Conclusion

In conclusion, sildenafil ameliorated colon injury and its associated oxidant and inflammatory features through multiple mechanisms. SILD down-regulated expression of TNF-α, NF-κB p65, and COX-2. In addition, SILD acted as an activator of Nrf-2/HO-1, and inhibitor of inflammation signaling pathways. Hence, sildenafil could serve as a promising candidate for the treatment of ulcerative colitis in humans. It may be nonessential to take sildenafil as a protective medicine, and its short term treatment could produce the desired coloprotective effect.

## Electronic supplementary material

Below is the link to the electronic supplementary material.

Supplementary file (DOCX 183 kb)

## Data Availability

The datasets analyzed during the current study are available from the corresponding author on reasonable request.

## Abbreviations

ARE: Antioxidant response elements
Cyclooxygenase-2: COX-2
GSH: Glutathione
H&E: Hematoxylin and eosin
HO-1: Heme oxygenase-1
MDA: Malondialdeyhde
NF-κB: Nuclear factor kappa B
NOx: Nitrite/nitrate
Nrf-2: Nuclear erythroid-related factor 2
PGE-2: Prostaglandin E2
SILD: Sildenafil
SOD: Superoxide dismutase
TNF-α: Tumor necrosis factor alpha
TXB2: Thromboxane B2
UC: Ulcerative colitis
