# Supplementary material for: The emerging coloprotective effect of sildenafil against ulcerative colitis in rats via exerting counterbalance between NF-κB signaling and Nrf-2/HO-1 pathway
Source: Inflammopharmacology. 2022 Jul 14;30(4):1351–62. doi: 10.1007/s10787-022-01016-9 (PMC9293796; doi:10.1007/s10787-022-01016-9)
Supplement: Supplementary file 1 — Supplementary file (DOCX 183 kb) [file 10787_2022_1016_MOESM1_ESM.docx]

**The emerging coloprotective effect of sildenafil against ulcerative colitis in rats via exerting counterbalance between NF-κB signaling and Nrf-2/HO-1 pathway**

**1. Aim of the preliminary study:**

1. Compare two different doses of sildenafil (SILD, 25-30 mg/kg) administered orally for 6 days to detect the most effective protective dose of SILD against acetic acid-induced ulcerative colitis. Then, the study was completed on the more efficient dose in alleviating the observed macroscopic disease activity index (DAI) and ulcer scores to determine the possible mechanisms by which SILD can reverse acetic acid-induced ulcerative colitis.
2. Determine the optimum time (3 or 6 days treatment) at which the effective dose of the drug, sildenafil could induce a marked alleviation in all measured markers of colon injury.

**2. Methods:**

**2.1. Experimental design**

Adult male SD rats weighing (250 ± 20 g) were divided into 5 groups (5 in each group).

- **Control group:** Rats were injected with 2 ml normal saline intrarectally at the induction day
- **Acetic acid (AA)** **group:** all rats were injected once with 2 ml acetic acid (3%) intrarectally, 2 days before sacrification to induce ulcerative colitis **(Sakthivel and Guruvayoorappan, 2013)**
- **SILD 25 + AA group**, received sildenafil (25 mg/kg, orally) for 6 days starting 3 days pre-injection of AA **(aim 1)**
- **SILD 30 + AA** **group**, received sildenafil (30 mg/kg, orally) for 6 days starting 3 days pre-injection of AA **(aim 1)**
- **SILD-t + AA group**, received sildenafil (25 mg/kg, orally), starting at the time of AA injection and continued for 3 days **(aim 2)**

All rats were deprived from food with free access to water 48 hrs before induction of colitis. One day 3 after induction of colitis, rats were anesthetized using thiopental (40 mg/kg, i.p.), after 1hr of the last dose administration. The entire colon was immediately excised and the colon length was measured and observed as an indirect marker of inflammation, then washed, opened longitudinally, scored for ulcerative colitis and photographed

**2.2. Determination of disease activity index (DAI)**

Animals were observed for body weight change, stool consistency and bleeding for 2 days post induction of colitis. The scores from these three parameters were summed to calculate the DAI, ranging from 0 (healthy) to 12 (maximal severity of colitis), using a protocol previously described by **(Cooper et al., 1993)** with little modification, as shown in **table 1**:

**Table 1. Scoring of disease activity index (DAI)**

| Weight loss | Stool consistency | Bleeding |
| --- | --- | --- |
| 0: < 1% | **0: normal** | **0: normal** |
| 1: 1-5% | **1: normal** | **1: occult blood (+)** |
| 2: 5-10% | **2: loose stool** | **2: occult blood (++)** |
| 3: 10-15% | **3: loose stool** | **3: occult blood (+++)** |
| 4: > 15% | **4: diarrhea** | **4: gross bleeding** |

**2.3. Determination of macroscopic ulcer score**

The ulcerative damage of the colon was assessed visually by the colon mucosal damage index (CMDI) scored on a scale of 0-10 according to a reported scoring system **(Bell et al., 1995)**. The following scores were applied: Grade 0: normal appearance; Grade 1: focal hyperemia, no ulcers; Grade 2: ulcer with no significant inflammation (hyperemia and bowel wall thickening); Grade 3: ulcer with inflammation at one site; Grade 4: two or more sites of ulceration and inflammation; Grade 5: major sites of damage extending > 1 cm along the length of the colon; and Grade 6-10: , if major sites of damage extending > 2 cm along the colon, the score was increased by 1 for each additional cm of involvement.

**3. Results:**

**Effect of sildenafil on DAI, colon length, and macroscopic ulcer score**

Acetic acid treatment caused a significant increase in DAI and macroscopic ulcer score with a significant reduction in colon length compared to control group. Meanwhile, other treated groups: SILD 25 + AA, SILD 30 + AA and SILD-t + AA, showed a marked decrease in intensity of DAI and ulcer score, with a more significant increase in colon length, compared to AA group **(Fig I)**, indicating the highest preference for the longer term (6 days) pretreatment with SILD (25 mg/kg) over the short term (3 days) post treatment with SILD-t (25 mg/kg), while no remarkable difference was observed between pretreatment with SILD (25 mg/kg) and the higher dose SILD (30 mg/kg) administered for 6 days, regarding improvement of all macroscopical features.

There are many sildenafil doses for rats, ranging from 0.4 mg/kg to 90 mg/kg **(Cadirci et al., 2011)**, meanwhile no adverse effect levels in were noticed in rats up to 60 mg/kg dose, while above 200 mg/kg dose in rats were limited by isolated deaths in repeated dose studies and a maximum nonlethal dose of 300 mg/kg in single dose studies in rats **(Abbott et al., 2004)**. Therefore, oral daily administartion of sildenafil (25 and 30 mg/kg) doses were chosen in this experiment for their safety and equivalence to plasma concentration nearly 20-fold the proposed clinical dose (100 mg) in humans **(Kim et al., 2005)**.

Since there was no difference in coloprotective effect between the two doses, as shown in **(Fig I),** the lower dose (25 mg/kg) was selected to proceed with study of pretreatment and treatment therapy with sildenafil. Moreover, previous studies confirmed the efficacy of 25 mg/kg oral dose of sildenafil against hypoxia- and monocrotaline-induced pulmonary hypertension **(Lee et al., 2010; Jasińska-Stroschein et al., 2013; Huang et al., 2022)**. In addition, this dose has been shown to be well tolerated with no hemodynamic instability **(Dias-Junior et al., 2005)**, geno- and cytotoxicity **(Lemus-Varela et al., 2006)**

**
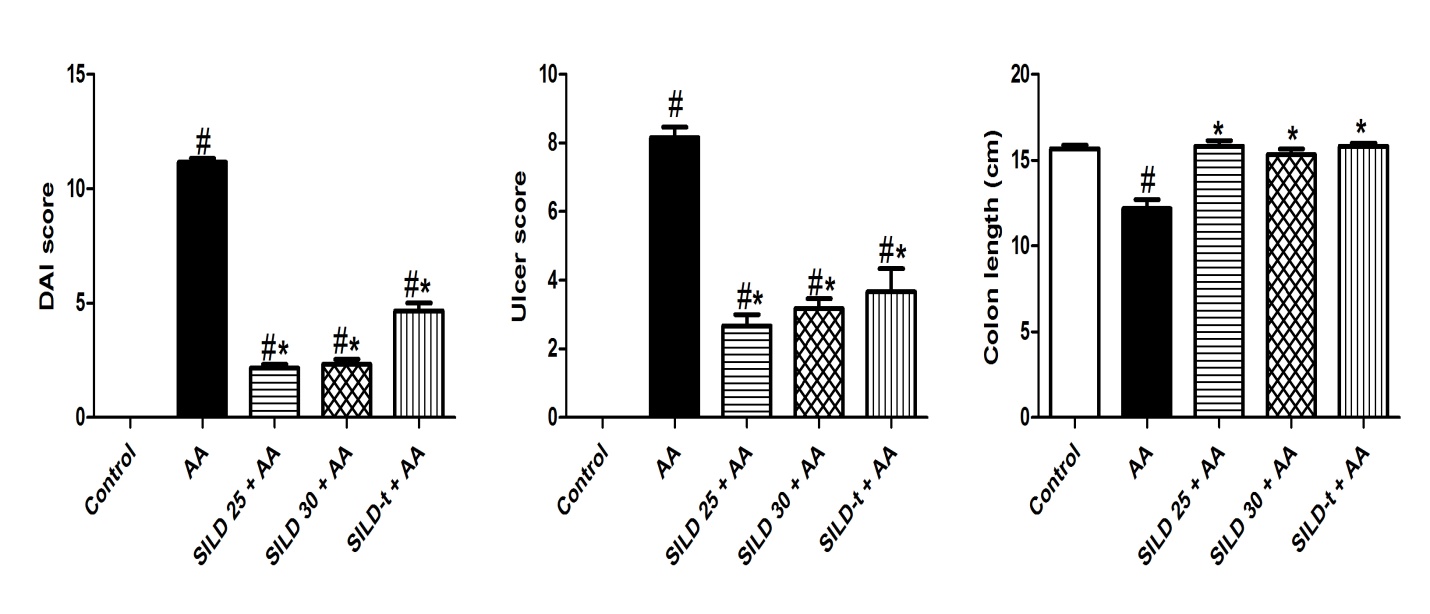
**

**Fig I. Effect of sildenafil on DAI, macroscopic ulcer score and colon length**

Data are expressed as mean ± SEM (n = 5) AA: Acetic acid; SILD: Sildenafil; DAI: disease activity index. Statistically significant: # p <0.05, compared to control group, * p <0.05, compared to AA group, ^@^ p <0.05, compared to SILD 25 + AA group, using one-way ANOVA followed by Tukey-Kramer multiple comparisons post hoc test.

**4. References:**

**Abbott, D.; P. Comby; C. Charuel; P. Graepel; G. Hanton; B. Leblanc; A. Lodola; L. Longeart; G. Paulus; C. Peters and J. Stadler (2004)**. Preclinical safety profile of sildenafil. International Journal of Impotence Research, 16: 498-504.

**Bell, C. J.; D. G. Gall and J. L. Wallace (1995)**. Disruption of colonic electrolyte transport in experimental colitis. Am J Physiol, 268: G622-630.

**Cadirci, E.; Z. Halici; F. Odabasoglu; A. Albayrak; E. Karakus; D. Unal; F. Atalay; I. Ferah and B. Unal (2011)**. Sildenafil treatment attenuates lung and kidney injury due to overproduction of oxidant activity in a rat model of sepsis: a biochemical and histopathological study. Clin Exp Immunol, 166: 374-384.

**Cooper, H. S.; S. N. Murthy; R. S. Shah and D. J. Sedergran (1993)**. Clinicopathologic study of dextran sulfate sodium experimental murine colitis. Lab Invest, 69: 238-249.

**Dias-Junior, C. A.; D. C. Souza-Costa; T. Zerbini; J. B. T. da Rocha; R. F. Gerlach and J. E. Tanus-Santos (2005)**. The Effect of Sildenafil on Pulmonary Embolism-Induced Oxidative Stress and Pulmonary Hypertension. Anesthesia & Analgesia, 101: 115-120.

**Huang, W.; N. Liu; X. Tong and Y. Du (2022)**. Sildenafil protects against pulmonary hypertension induced by hypoxia in neonatal rats via activation of PPARγ‑mediated downregulation of TRPC. Int J Mol Med, 49: 19.

**Jasińska-Stroschein, M.; J. Owczarek; A. Łuczak and D. Orszulak-Michalak (2013)**. The beneficial impact of fasudil and sildenafil on monocrotaline-induced pulmonary hypertension in rats: a hemodynamic and biochemical study. Pharmacology, 91: 178-184.

**Kim, E. J.; J. W. Seo; J. Y. Hwang and S. S. Han (2005)**. Effects of combined treatment with sildenafil and itraconazole on the cardiovascular system in telemetered conscious dogs. Drug Chem Toxicol, 28: 177-186.

**Lee, D. S.; Y. K. Kim and Y. W. Jung (2010)**. Simvastatin, sildenafil and their combination in monocrotaline induced pulmonary arterial hypertension. Korean Circ J, 40: 659-664.

**Lemus-Varela, M. L.; A. Sola; B. C. Gómez-Meda; A. L. Zamora-Perez; M. L. Ramos-Ibarra; C. M. Batista-González and G. M. Zúñiga-González (2006)**. Oral sildenafil citrate lacks genotoxicity and cytotoxicity in a primate model: Callithrix jacchus. J Perinatol, 26: 423-427.

**Sakthivel, K. M. and C. Guruvayoorappan (2013)**. Amentoflavone inhibits iNOS, COX-2 expression and modulates cytokine profile, NF-κB signal transduction pathways in rats with ulcerative colitis. Int Immunopharmacol, 17: 907-916.
